# Next-generation resequencing the complete mitochondrial genome of Japanese quail (*Coturnix japonica*)

**DOI:** 10.1080/23802359.2016.1258343

**Published:** 2017-01-04

**Authors:** Gang Liu, Yingyin Zhang

**Affiliations:** Department of Biology, School of Life Science, Anhui Medical University, Hefei, P. R. China

**Keywords:** NGS technologies, Japanese quail, *Coturnix japonica*, complete mitochondrial genome sequences

## Abstract

As the development of the new generation of sequencing (NGS) technologies, it has been used for standard sequencing applications more and more popular. We used NGS technologies to resequence the complete mitochondrial genome of Japanese quail. The complete mitochondrial genome of Japanese quail is a 16,668 bp circular molecule, which contains 37 typical mitochondrial genes (13 protein-coding genes, 2 rRNAs, and 22 tRNAs) and a 1156 bp D-loop. Its gene arrangement pattern is identical with typical other Galliformes. All protein-coding genes start with an ATG codon except *COI*, which start with GTG. TAA is the most frequent stop codon, although *ND2* end with TAG, and *COI* and *ND6* end with AGG, *COIII* and *ND4* end with TGC. The mtDNA sequence contains 12S rRNA and 16S rRNA of rRNA. Except for *tRNA^Ser(AGY)^* and *tRNA^Leu(CUN)^* without the dihydrouridine (DHU) arm, all tRNAs could be folded into canonical cloverleaf secondary structures. *Coturnix japonica* has close relative with *C. chinensis*.

The new generation of sequencing (NGS) technologies has provided unprecedented opportunities for high-throughput functional genomic research (Morozova & Marra 2008). Comparing to Sanger sequencing, NGS has been made in terms of speed, read length, and throughput, along with a sharp reduction in per-base cost, it has been more and more popular (Morozova & Marra 2008).

Japanese quail, *Coturnix japonica* belongs to the family Phasianidae and the order Galliformes, which breeds in eastern Asia, wintering in southern China and Southeast Asia. The Japanese quail is similar in appearance and has closely related to the Common quail, *C. coturnix*. Some authors think that these quail will interbreed and produce fertile hybrids (Harper 1986; Johnsgard 1988). In this study, we resequenced and characterized the complete mitochondrial genome used NGS technologies, and expected that it could provide more molecular data for the genetic studies of Japanese quail.

The blood sample of Japanese quail was acquired from the specimen storeroom of the laboratory in School of Life Science, Anhui Medical University (Sample codes are AMHU-AN20151023) and were stored at −80 °C. The blood sample was collected using non-invasive methods from a poultry farm in Nanjing City, no animal was killed for the purpose of the experiment. The mtDNA was sequenced by Sangon Biotech Co., Ltd. (Shanghai, China) using next-generation sequencing technology.

The length of the complete mtDNA sequence is 16,698 bp, is longer than the species which has been published in 2001 (accession no. AP003195, 16 697 bp in length) (Nishibori et al. 2001). Comparing to accession no. AP003195 sequence, 29 variable sites were observed, 11 sites were in the D-loop region, 16 sites were in the protein-coding gene (*ND2*, *COI*, *COIII*, *ND3*, *ND4L*, and *Cyt b*) regions, and two sites were in tRNA regions (*tRNA^Asp^* and *tRNA^Glu^*). The overall base composition for the mtDNA sequence is as follows: A, 31.1%, C, 31.3%, G, 13.1%, and T, 24.5%. A + T content (55.6%) is higher than C + G content (44.4%), similar to other aves species (Liu et al. 2013). The length of intergenic spacer sequences is 46 bp at 17 locations and the overlapping bases are 34 bp existing in 10 regions (Table 1). Among the the 13 PCGs, the longest one is *ND5*, and the shortest is *ATP8*, all protein-coding genes start with an ATG codon except for *COI,* which starts with GTG. TAA is the most frequent stop codon, although *ND2* end with TAG, and *COI* and *ND6* end with AGG, *COIII* and *ND4* end with TGC (Table 1). The new mtDNA sequence contains 12S rRNA and 16S rRNA of rRNA, which are located between *tRNA^Phe^* and *tRNA^Leu^*, separated by tRNAVal. The 12S rRNA is 974 bp long and the 16S rRNA is 1615 bp in length. All tRNA genes possess the typical clover leaf secondary structure except for *tRNA^Ser(AGN^*^)^ and *tRNA^Leu(CUN^*^)^, which lacks a dihydroxyuridine (DHU) arm. The non-coding regions include a D-loop and a few intergenic spacers. The D-loop is located between *tRNA^Phe^* and *tRNA^Glu^*, and is 1156 bp long.

Phylogenetic trees were estimated using ML and BI methods, based on the complete mtDNA of eight Galliformes species, and corresponding *Aix galericulata* (KF437906) sequence was used as an outgroup, sharing similar topologies and high node support values (Figure 1). *Coturnix japonica* has close relative with *C. chinensis*.

**Table 1.** Organization of the mitochondrial genome of *Coturnix japonica*.

Gene	Direction	Nucleotide no	Size	Spacer (+) or Overlap (−)	Start codon	Stop codon
CR	F	1–1156	1156	0		
tRNA^phe^	F	1157–1223	67	0		
rRNA-Ssu	F	1224–2197	974	0		
tRNA^val^	F	2198–2268	71	0		
rRNA-Lus	F	2269–3883	1615	0		
tRNA^leu^	F	3884–3957	74	8		
ND1	F	3966–4940	975	0	ATG	TAA
tRNA^ile^	F	4941–5011	71	6		
tRNA^gln^	R	5017–5087	71	5		
tRNA^met^	F	5087–5155	69	−1	ATG	TAG
ND2	F	5156–6196	1041	−1		
tRNA^trp^	F	6196–6270	76	5		
tRNA^ala^	R	6276–6344	69	2		
tRNA^asn^	R	6347–6419	73	0		
tRNA^cys^	R	6420–6485	66	−1		
tRNA^tyr^	R	6485–6555	71	1	GTG	AGG
COI	F	6557–8107	1551	−9		
tRNA^ser^(UCN)	R	8099–8173	75	2		
tRNA^asp^	F	8176–8244	69	1		
COII	F	8246–8929	684	1	ATG	TAA
tRNA^lys^	F	8931–8998	68	1		
ATP8	F	9000–9167	168	−10	ATG	TAA
ATP6	F	9158–9841	684	−1	ATG	TAA
COIII	F	9841–10626	786	−1	ATG	TGC
tRNA^gly^	F	10626–10693	68	0		
ND3	F	10694–11045	352	1	ATG	TAA
tRNA^arg^	F	11047–11115	69	0		
ND4L	F	11116–11412	297	−7	ATG	TAA
ND4	F	11406–12785	1380	−2	ATG	TGC
tRNA^his^	F	12784–12852	69	1		
tRNA^ser^ (AGY)	F	12854–12922	69	1		
tRNA^leu^(CUN)	F	12924–12994	71	0		
ND5	F	12995–14815	1821	−1	ATG	TAA
Cytb	F	14815–15957	1143	3	ATG	TAA
tRNA^thr^	F	15961–16030	70	2		
tRNA^pro^	R	16033–16102	70	5		
ND6	R	16108–16629	522	1	ATG	AGG
tRNA^glu^	R	16631–16698	68			

**Figure 1.**
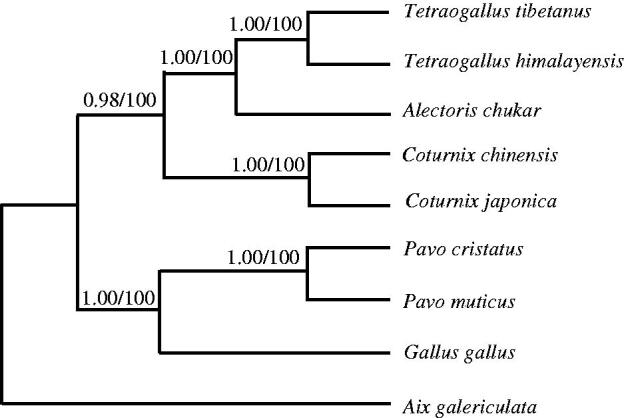
Phylogenetic relationships among the eight Galliformes’ species based on complete mtDNA sequences. Numbers at each node are Bayesian posterior probabilities (left) and maximum likelihood bootstrap proportions (estimated from 100 pseudoreplicates) (right). The accession number in GenBank of eight Galliformes in this study: *Gallus gallus* (GU261698), *Pavo muticus* (EU417811), *P. cristatus* (KF444060), *C. chinensis* (AB073301), *Alectoris chukar* (FJ752426), *Tetraogallus himalayensis* (KR349185), *T. tibetanus* (KF027439).

## Nucleotide sequence accession number

The complete mtDNA sequence of Japanese quail has been assigned with GenBank accession number KX712089.
